# A Ten-year Report of Drug and Poison Information Center in Mashhad, Iran 2007-2017

**DOI:** 10.22037/ijpr.2020.112228.13617

**Published:** 2021

**Authors:** Anoosheh Maruzi, Sara Sabbaghian-Tousi, Gholamreza Karimi, Roya Jabbari, Sepideh Elyasi

**Affiliations:** a *Department of Clinical Pharmacy, School of Pharmacy, Mashhad University of Medical Science, Mashhad, Iran. *; b *Department of Community Health and Epidemiology, College of Medicine, University of Saskatchewan, Saskatoon, SK, Canada. *; c *Department of Biostatistics, School of Health, Mashhad University of Medical Science, Mashhad, Iran. *; d *Pharmaceutical Research Center, Faculty of Pharmacy, Mashhad University of Medical Sciences, Mashhad, Iran. *; e *Research and Development Center, Food and Drug Administration, Mashhad University of Medical Sciences, Mashhad, Iran.*

**Keywords:** Drug information center, Poison information center, Epidemiology, DPIC

## Abstract

The Mashhad drug and poison information center (MDPIC) was officially established in 2000 to provide up-to-date information on medications. The objective of this study is to provide an epidemiologic profile of drug inquiry and poisoning-related phone calls to MDPIC from 2007 to 2017. This article is a descriptive retrospective study in which all inquiries about drugs and poisoning cases received by MDPIC, from 1^st^ January 2007 to 31^st^ December 2017, were retrieved from its database for analysis. A total of 100997 cases were analyzed. The most frequent calls were from individuals in the age group of 18 to 60 years old (70.21%). The majority of callers were women (73.08%). The public made 95.11% of calls, and 4.89% were related to health care professionals. The queries were mainly related to therapeutic uses of drugs (24.03%), followed by adverse drug reactions (18.96%). Given that 99.23% of calls were related to drug information inquiries, the most common drugs questioned about were antimicrobial (12.3%) and vitamin and minerals (10.76%), whereas 0.77% of calls were about poisoning and the majority of them were due to drugs poisoning. Micromedex® was the most commonly used reference to answer the inquiries. This report shows an updated epidemiological evaluation on recorded calls in the drug and poison information center in Mashhad. Since there is no other similar report, this can provide valuable information on the trend of drug usage and may guide further strategies in giving proper information to public and health centers.

## Introduction

Recently, the advances of medical sciences accompanied by the complexity of drug therapies have raised a need for the establishment of centers providing authentic scientific information in this respect (1). Moreover, the drug information provided by the health professionals in a face-to-face consultation with patients is often suboptimal (2, 3). For these reasons, as well as the inadequate awareness of the poisoning management among the medical team, the drug and poison information centers (DPIC) have been established with the aim of promoting rational use of drugs in the healthcare system (4).

These centers are specialized units which provide the health professionals and general population with accurate, evidence-based, and up-to-date information on drugs and poisons (5-9). Detection and prevention of adverse drug reactions (ADRs), and medication errors, and also promoting rational use of drugs are some of the benefits provided by DPIC, which in turn improve national drug policy and its standardized indicators (9-13). Moreover, DPICs play a critical role in reducing medical expenses by preventing drug misuses. Poison information services are highly cost-effective, as timely information may reduce healthcare expenditures by decreasing unnecessary admissions and prolonged hospitalization. Consequently, these centers may improve health outcomes and reduce countrywide healthcare costs (14-16).

The first drug and poison information center was established in the United States in 1962. Subsequently, other DPICs have been installed to offer evidence-based drug and poison information to healthcare professionals and the public worldwide (17-19). Given that healthcare services are less available in developing countries, the existence of DPICs seems beneficial in such countries (5). The first national DPIC in Iran was established in Tehran in 1997 as a part of the food and drug organization of Iran under the ministry of health and medical education (MOH) as the national DPIC. Afterward, by the co-operation of the national DPIC, other DPICs were established countrywide. Among these centers were Mashhad drug and poison information center which was established in 2000 (9, 20). At MDPIC, trained pharmacists are responsible for answering inquiries regarding drug and poison information under the supervision of a clinical pharmacist. MDPIC’s services are available to all citizens free of charge. Currently, this center provides a 14 h service (8 a.m. to 10 p.m.) in three working shifts every day except for official holidays.

In an effort to increase public education on drug safety issues, while considering that pharmaceutical products are the leading causes of poisoning in Iran, the introduction of DPICs to the Iranian population seems to be advantageous (5). The statistical reports of DPICs play a pivotal role in the improvement of managerial and political aspects of public health by raising awareness about the services offered by DPIC and therefore promoting safe and effective drug therapy and improve patient outcomes. Adding to that, the results of this study may help to improve the quality of services provided by our center. The objective of the present study was to provide an epidemiologic profile of the recorded inquiries received by the MDPIC from 2007 to 2017. 

## Experimental

This was a descriptive retrospective study that reviewed recorded phone calls in MDPIC in Iran from 1^st^ January 2007 to 31^st^ December 2017.

A printed form is used to record information regarding every call received. Collected data included date and time of calls, the identity of the pharmacists who answered the calls, gender and age of the callers accompanied by a brief summary of the asked question, including the type of the inquiries (*e.g.*, drug identification, the therapeutic use of drugs, adverse drug reactions (ADRs), drug interactions, poisoning- related calls, *etc.*), category of drugs, type of poisons in poisoning cases and medical references used to answer the inquiries. After recording data on the printed version of the forms, each staff transfers the written information into an electronic form. The electronic data was extracted from the database and cases with incomplete data were excluded. Subsequently, statistical analysis was performed using Microsoft Excel (Microsoft Corp., Redmond, WA, USA). The results are presented as frequencies and percentages.

The identities of the callers were not recorded as part of the data collection process in order to address ethical consideration.

## Results

The total number of calls documented at the MDPIC during 2007-2017 was 100997. 

The distribution of age and gender among patients whom questions were related to them are outlined in Table 1. As the data shows, the majority of the callers (73.08%) were female. Most of patients who contacted with MDPIC was in the age group of 18-60 years old (70.21%). The mean age of the callers is 31 years old. Most calls were made by the patients and the patients’ relatives (95.11%) followed by the pharmacists (2.87%), and general practitioners (1.06%).

**Figure 1:**
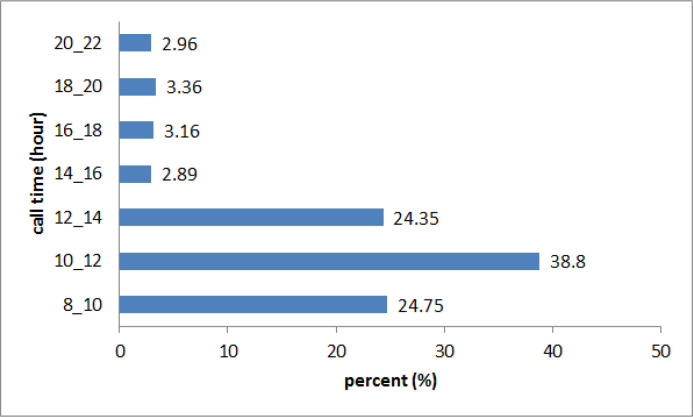
Time of calls in MDPIC report in 2007-2017

According to Figure 1, the majority of the calls were received between 10 a.m. and 12 a.m. (38.8%).

**Figure 2:**
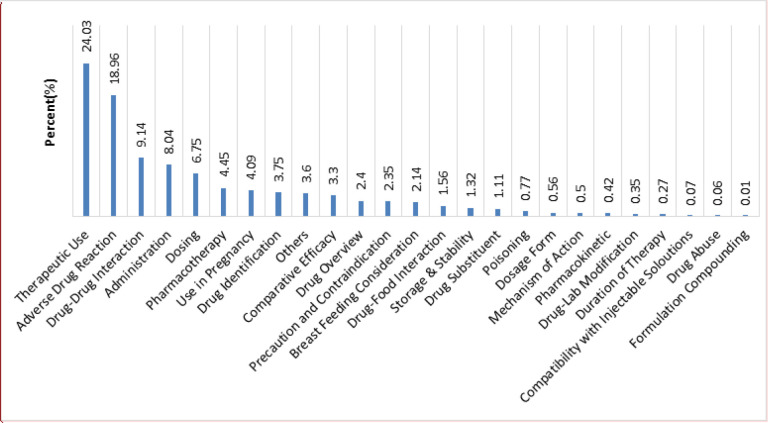
Type of questions to the MDPIC from January 2007 to September 2017

Figure 2 shows the distribution of various requested inquiries classification. According to the results, the callers most frequently requested information about the therapeutic use (24.03%), ADR (18.96%), drug-drug interaction (9.14%), and administration (8.04%), respectively.

**Figure 3:**
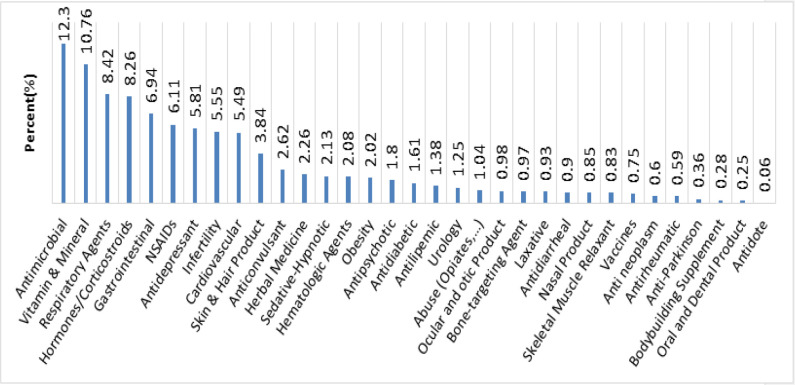
The medications category distribution was questioned in MDPIC from 2007 to 2017

Figure 3 indicates that the questions were mostly related to antimicrobial (12.3%) and vitamin and mineral (10.76%). On the other hand, the least frequent inquiries were related to antidote (0.06%). 

**Figure 4:**
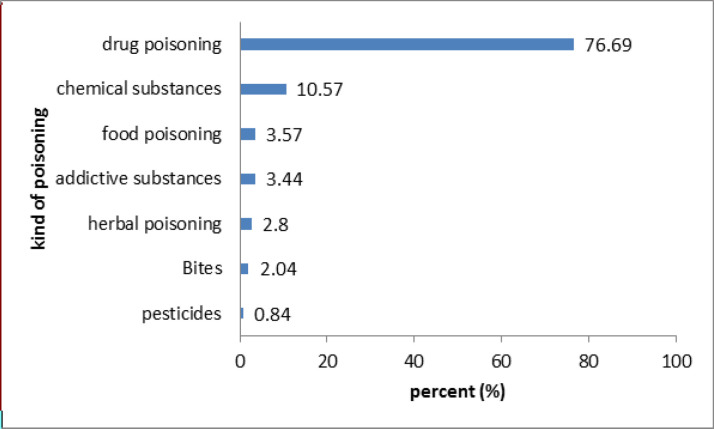
Kind of poisoning in MDPIC report in 2007-2017

Figure 4 demonstrates that the most frequent toxicities arose from pharmaceutical agents (76.69%), followed by chemical poisoning (10.57%). Whereas the least common cause of the poisoning cases were related to pesticides (0.84%).

According to Table 2, Micromedex® (61.71%) was the most commonly used reference to answer the inquiries.

## Discussion

In the present study, Mashhad’s DPIC activity for ten years was evaluated. Overall, 100997 calls were received by MDPIC since 2007 until 2017. The number of calls recorded in 2017 was 17781, which shows approximately a 10 times increase in the number of calls compared with 1668 calls received in 2007. The reason for such an increase may be the completion of MDPIC’s staff and equipment through these years and increased public awareness about services provided by MDPIC. Although the number of calls has significantly increased during these ten years, it still seems to be low considering the population who are under MDPIC supervision, based on the latest report available on the health information exchange (HIX) system (about 5’087’992 persons) (21). Thus, new policies regarding introducing MDPIC to the public should be implemented. 

Majority of the calls (95.11%) were made by the general public, and 4.89% of the calls were related to the health care professionals. This pattern is consistent with other reports in Iran (5, 11 and 20). The low rate of health care professionals’ engagement with DPICs indicates that there is a need for implementation of informing programs to improve the health care professionals’ awareness about DPIC’s services. Among health care professionals, pharmacists made the majority of the calls (58.69%), followed by general practitioners (21.68%). This trend is similar to other studies in Iran (5, 11 and 20). More advertisement of DPICs in pharmacies may be the reason for this finding. 

Queries were predominantly made by females (73.08%), which agrees with the findings of other studies conducted in Iran, Finland, and the Netherlands (5, 8, 9, 11, 20, 22 and 23). This may be the case because women have more free time in Iran as well as greater responsibility for family health. Moreover, most of the calls came from the age group of 18-60 years old, which is in line with previous studies in Iran (5, 8, 11 and 20). It seems that despite the excessive use of medication in the elderly population, they are not familiar with this telephone service and also, their pharmaceutical questions are may be asked from DPIC by their children. 

Most of the received inquiries were related to therapeutic use (24.03%), followed by ADR (18.96%). These findings are in line with the previous reports in Iran, which could be due to the inappropriate drug consultation of physicians and community pharmacists to the patients in these areas (5, 9, 11, 20 and 23). Therefore, the drug information centers may have a critical role in improving patients’ awareness about their medicine. Moreover, providing the patients with accurate information regarding adverse drug reactions may deter undesirable health and economic consequences of ADRs, as they are the fourth to the sixth leading cause of death in the United States with an annual cost of $1.5-$4 billion to the health care system. According to the pharmacovigilance studies, ADRs are responsible for approximately 2.9-5% of hospital admissions, as well as more than 100,000 deaths per year in the United States (11, 24 and 25).

According to a worldwide survey on antibiotic usage, antibiotics account for the largest proportion of drug expenditure in developing countries, including Iran (26). Moreover, 58% of prescriptions in Iran contain at least one antibiotic (27). Given the overuse of antibiotics in Iran, the distribution of inquiries regarding antimicrobials (12.3%) in this study is consistent with the countrywide pattern of antibiotic use. Vitamin and minerals are in second place regarding the number of asked inquiries. This pattern is in accordance with other studies in Iran, which indicates a need to implement policies to increase public information about this category of drugs (5, 20). Such implementations may include designing scientific pamphlets providing the general public with practical information on vitamins and minerals. 

According to the two surveys conducted in Imam Reza hospital, which is the only tertiary referral center for intoxicated patients in Khorasan Razavi province, the total number of hospital-referred poisoned patients evaluated during 1993-2000 and 2004-2013 were 71’589 and 49’189 cases, respectively (28, 29). In this study, the total number of poisoning cases called the center from 2007 to 2017 was 792, which is very low comparing to the total number of poisoning cases mentioned in the stated studies. This low rate of received poisoning cases in MDPIC is similar to the previous studies on other DPICs in Iran (9, 20, 30 and 31). This pattern may be related to the inadequate public awareness of DPIC as a reliable source of information and guidance for poisonings. Taking into account that DPICs have a crucial role in the management of poisoning cases, especially in early and pre-hospital phases, more efforts should be made to improve the laypersons’ education about these centers. 

Among the poisoning cases recorded in Mashhad DPIC, pharmaceutical agents were responsible for the majority of the cases, which is in line with other reports in Iran (5, 9, 20, 29 and 30). This trend may be due to the high prevalence of self-medication (53%) in Iran, which is relatively more than in other countries in the world (32). For example, in the 2015 Annual Report of the American Association of Poison Control Centers’ National Poison Data System (NPDS), analgesics (11.1%), household cleaning substances (7.54%), cosmetics/personal care products (7.41%), sedatives/hypnotics/antipsychotics (5.83%), and antidepressants (4.58%) were the most common substance classes that patients reported as poisoning cases (33). A report from the poison information center of Japan on accidental poisoning of children reported that most of the cases were with household products (34). Currently, the dispensing of prescription-only drugs without a prescription is prohibited by law in Iran. Nevertheless, the practice of self-prescription is already widespread in Iran because the law is not strictly run on the pharmacies (35). Therefore, there is a need for appropriate planning and implementing policies to address this problem.Moreover, worth mentioning that despite the widespread use of pesticides, we did not have too many questions about them. It may be due to their wide usage by the rural population and limited knowledge of them about DPIC center. 

MDPIC aims to provide the public and health care professionals with evidence-based information in order to reduce medication errors, adverse reactions and prevent wasting resources. In an effort to deliver reliable drug and poison information, valid references such as Micromedex® and UptoDate® are used in MDPIC. The key resource used by our center to answer questions regarding drug information was Micromedex®, which is also used widely in other drug information centers in Iran and United States (9, 19, 20 and 36). A Canadian study aimed to determine users’ preferences among the most commonly used online drug information databases has shown that the Lexi-Comp® was of higher preference than Micromedex® (37). Lexi-Comp® provides the drug information available in UptoDate®. In a study on the performance of DPIC in southwestern Iran, most questions were answered by using UptoDate® (23). As regards the low rate of using UptoDate® in this study (6.32%), it should be mentioned that our center has started using this valuable evidence-based online resource since 2016. According to the recorded data in our center, the rate of usage of this database in 2016 and 2017 was 20% and 18%, respectively. Therefore, the absence of this online resource during 2007-2015 may account for its low rate of usage through this ten-year survey. Moreover, to address poisoning cases, our staff utilize poisindex® which is used in other poison information centers as well (14, 35).

**Table 1:** Characteristics of patients whom questions were related to them in the MDPIC report in 2007-2017

**Variables**	**N (%)**
**Gender of callers**	
Female	70504 (73.08)
Male	25967 (26.92)
**Age of callers**	
<2	8976 (9.36)
2-12	10426 (10.88)
12-18	2248 (2.35)
18-30	24470 (25.53)
30-40	22687 (23.67)
40-60	20144 (21.01)
>60	6907 (7.2)
**Identity of callers**	
Patients and patient's relatives	96055 (95.11)
Pharmacist	2900 (2.87)
General Practitioners	1074 (1.06)
Specialists	206 (0.2)
Other health care professionals	762 (0.76)

**Table 2:** References and sources for answering in MDPIC report in 2007-2017

**References and sources**	**N (%)**
Micromedex, Drugdex software	81731 (61.71)
Other internet resources and sites	19444 (14.68)
Iranpharma	8908 (6.73)
Up to Date software	8368 (6.32)
Drugs in Pregnancy and Lactation (Briggs) book	4430 (3.34)
Herbal Medicines books	1866 (1.41)
Drugs.com site	1823 (1.38)
Martindale book	1580 (1.19)
Drug Facts and Comparisons book	1418 (1.07)
Drug Brochures	817 (0.62)
Poisindex	756 (0.57)
PDR	351 (0.27)
USPDI	209 (0.16)
Handbook on Injectable Drugs	208 (0.16)
NFI	188 (0.14)
AHFS Drug Information	178 (0.13)
Articles published in journals (indexed in valid databases)	71 (0.05)
Applied Therapeutics book	62 (0.05)
Herbal Therapy and Supplement	12 (0.01)
Goodman&Gilmans\Katzung book	9 (0.01)
Hadad-Winchester Clinical Management of poisoning and Drug	5 (0.003)
Remington	4 (0.003)
The 5 minutes Toxicology Consult	1 (0.0007)
Harrison's Internal Medicine	1 (0.0007)

## Conclusion

According to the received queries, patients have numerous unmet drug information needs in Iran. Considering the pivotal role of DPICs in providing accurate drug and poison information to laypersons, this practice can significantly improve health education. From an economic perspective, DPICs may contribute to decreasing unnecessary expenditures by preventing adverse reactions, unnecessary hospitalization, and so forth. Moreover, based on the low rate of received calls from health care professionals, DPIC services should be introduced more in this respect, for it can help with preventing medication errors. Finally, although the number of queries is increasing when compared annually, more efforts should be made to educate the public about these centers’ services. It is suggested that the government should supply more funds to advertise DPICs through society.
